# Diadenosine tetraphosphate modulated quorum sensing in bacteria treated with kanamycin

**DOI:** 10.1186/s12866-023-03113-3

**Published:** 2023-11-17

**Authors:** Xia Ji, Ruojing Yu, Meilian Zhu, Cuilin Zhang, Libin Zhou, Tianshu Cai, Weiwei Li

**Affiliations:** 1 School of Life Science, Huizhou University, Huizhou, 516007 China; 2 Huizhou Health Sciences Polytechnic, Huizhou, 516025 China

**Keywords:** Kanamycin, Diadenosine tetraphosphate, Biofilm, *apaH*, Quorum sensing

## Abstract

**Background:**

The dinucleotide alarmone diadenosine tetraphosphate (Ap4A), which is found in cells, has been shown to affect the survival of bacteria under stress.

**Results:**

Here, we labeled Ap4A with biotin and incubated the labeled Ap4A with the total proteins extracted from kanamycin-treated *Escherichia coli* to identify the Ap4A binding protein in bacteria treated with kanamycin. Liquid chromatography‒mass spectrometry (LCMS) and bioinformatics were used to identify novel proteins that Ap4A interacts with that are involved in biofilm formation, quorum sensing, and lipopolysaccharide biosynthesis pathways. Then, we used the *apaH* knockout strain of *E. coli* K12-MG1655, which had increased intracellular Ap4A, to demonstrate that Ap4A affected the expression of genes in these three pathways. We also found that the swarming motility of the *apaH* mutant strain was reduced compared with that of the wild-type strain, and under kanamycin treatment, the biofilm formation of the mutant strain decreased.

**Conclusions:**

These results showed that Ap4A can reduce the survival rate of bacteria treated with kanamycin by regulating quorum sensing (QS). These effects can expand the application of kanamycin combinations in the treatment of multidrug-resistant bacteria.

**Supplementary Information:**

The online version contains supplementary material available at 10.1186/s12866-023-03113-3.

## Background

At present, antimicrobial resistance (AMR) is a global public health crisis. Kanamycin is a classical aminoglycoside antibiotic and has a good bactericidal effect and a very low rate of drug-resistance [1]. A high dose of kanamycin can even kill persistent or resistant bacteria that cannot be treated with other antibiotics [1, 2]. Recently, aminoglycoside antibiotics have been used in combination with other compounds in the clinic, which not only improved the bactericidal effects but also reduced adverse reactions [3, 4]. Thus, kanamycin has received renewed attention.

Diadenosine tetraphosphate is a dinucleotide metabolite that is found in both prokaryotes and eukaryotes. It is one of the most studied members of the NpnN family and consists of two adenosine moieties bridged by a polyphosphate chain of 2–7 phosphate groups [5]. Since the NpnN family was initially discovered more than 50 years ago, their function has long been thought to be secondary messengers or alarmones under stress [6–10]. Ap4A is mainly produced by aminoacyl-tRNA synthetases upon oxidative stress in *Escherichia coli* [11, 12] and degraded by Ap4A hydrolases, such as *apaH* [13, 14]. The *apaH* mutant strain could regulate bacterial biofilm formation [15], heat shock response sensitivity, oxidative stress, and bacterial drug resistance [16, 17]. Furthermore, the concentration of Ap4A in *Escherichia coli* was increased when bacteria were treated with kanamycin, and the increase in intracellular Ap4A could enhance the ability of kanamycin to kill many antibiotic-resistant bacteria, such as *E. coli*, *Acinetobacter baumannii*, and *Pseudomonas aeruginosa* [18].

In this study, we investigated the Ap4A binding proteins in kanamycin-treated bacteria by incubating biotin-labeled Ap4A with the proteins of treated *E. coli*; analysis was performed by liquid chromatography‒mass spectrometry (LCMS). According to the GO function, KEGG signaling pathway and protein interaction network analyses, 10 proteins were found to be enriched in signaling pathways related to biofilm formation, quorum sensing and LPS biosynthesis proteins. Bacteria have a quorum sensing (QS) mechanism that uses intercellular communication to promote bacterial biofilm formation and motility [19–23]. The biofilm formation of bacteria is associated with pathogenicity and virulence factors and causes fatal infections and antibiotic resistance [24, 25]. To investigate the effect of Ap4A on bacterial quorum sensing (QS), we used the *apaH* mutant strain of *E. coli* MG1655 to increase the concentration of Ap4A in bacteria and found that the biofilm formation ability of the *apaH* mutant strain was significantly reduced compared with that of the wild-type strain in the presence of kanamycin. At the same time, the accumulation of Ap4A in kanamycin-treated bacteria also changed the swarming motility ability of bacteria. Our findings show that Ap4A can affect the formation and movement of bacterial biofilms through quorum sensing, which further inhibits the viability of bacteria under adverse conditions. According to this mechanism, not only can the targets of kanamycin treatment be broadened but these findings also have important significance in alleviating the crisis of bacterial resistance.

## Results

### Enrichment of Ap4A-specific binding proteins

**Fig. 1:**
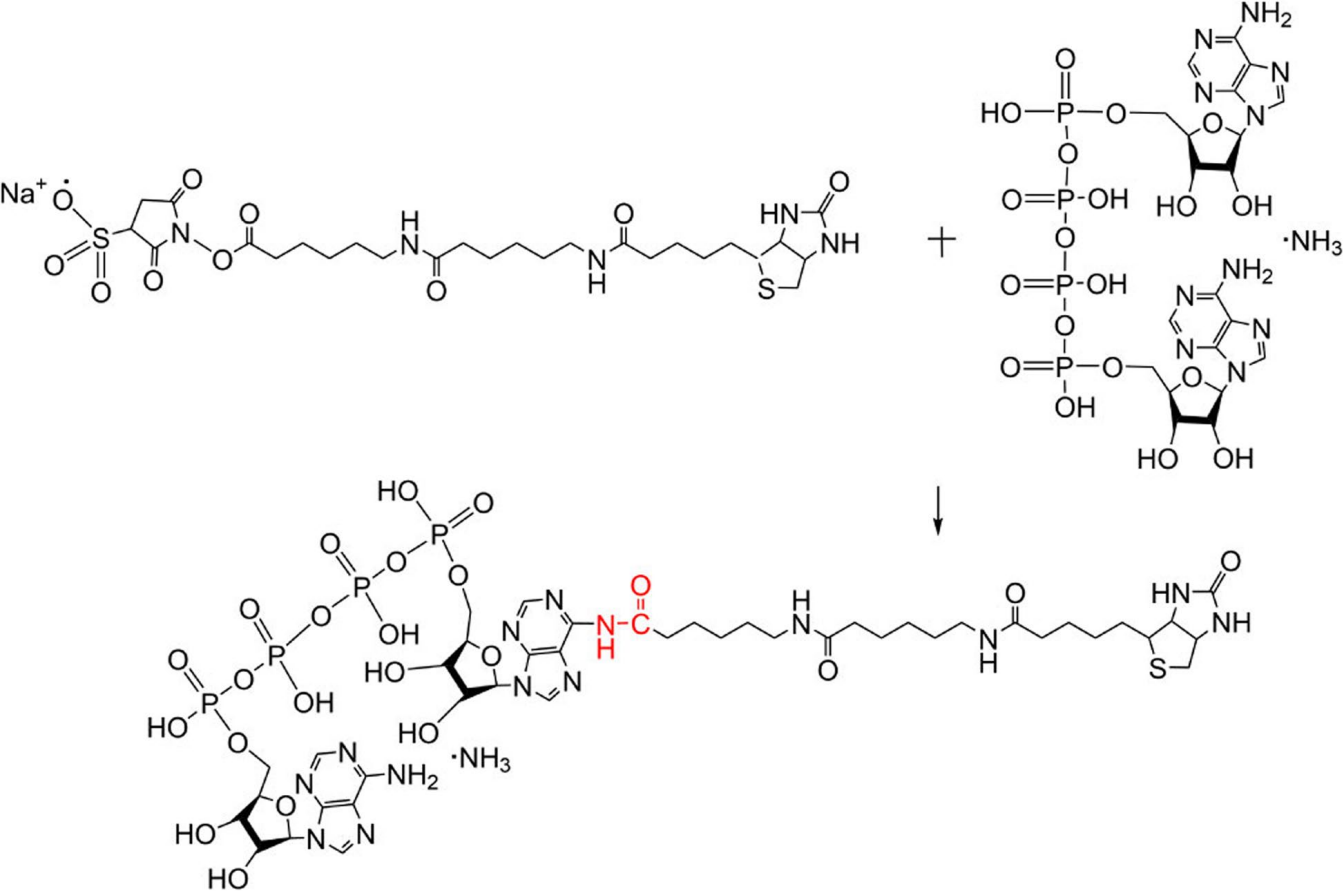
Biosynthetic protocol for the production of the biotin-conjugate product Ap4A-biotin

**Fig. 2:**
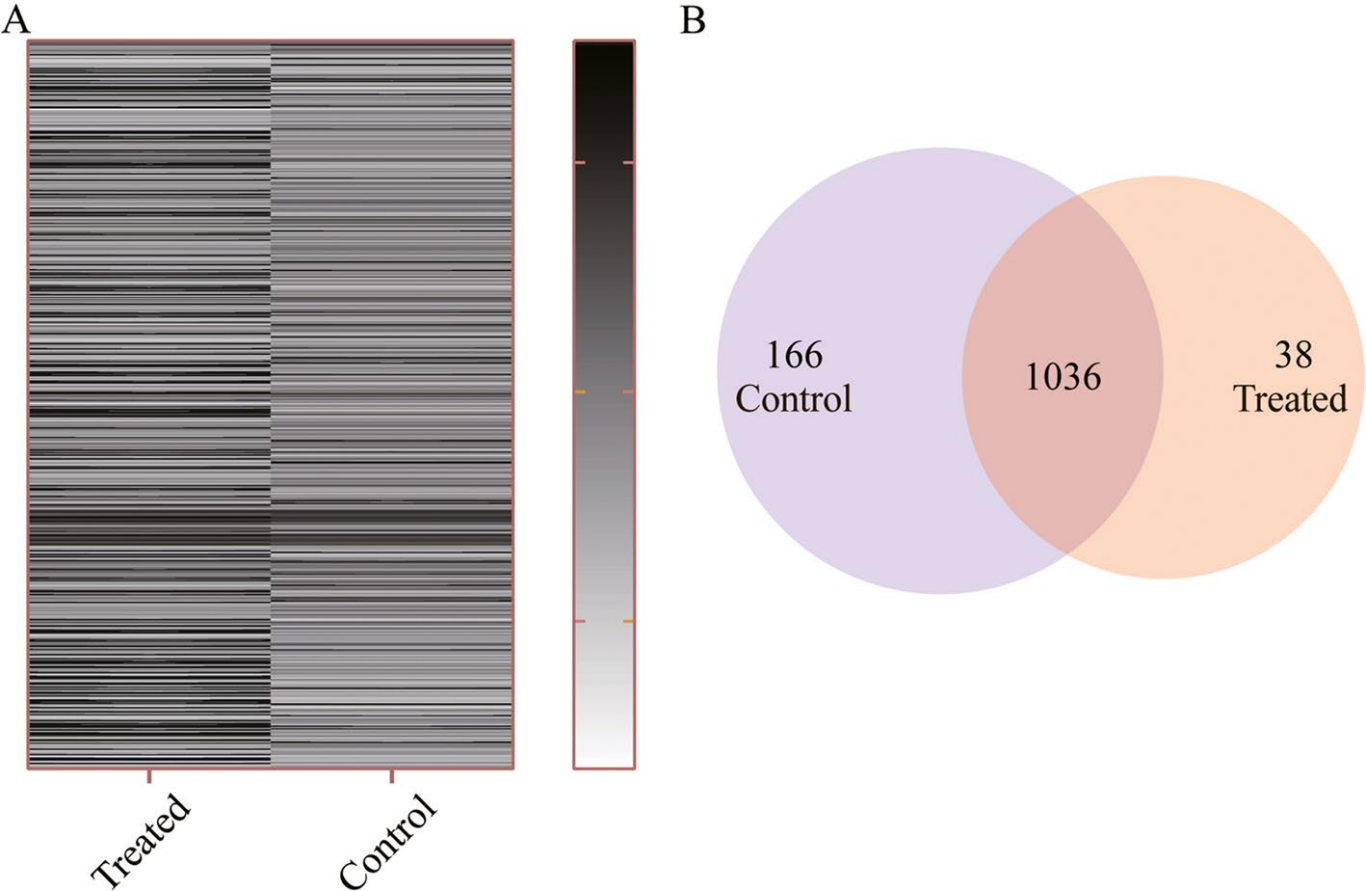
The Ap4A-interacting protein was screened in *Escherichia coli* K12-MG1655 treated with 100 μg/ml kanamycin by MALDI-TOF–MS proteomics. **A** Heatmap of Ap4A binding proteins in kanamycin-treated and untreated *E. coli* K12-MG1655. **B** Venn diagram of Ap4A binding proteins in kanamycin-treated and untreated *E. coli* K12-MG1655

Based on the previous research on Ap4A binding proteins [26, 27], we prepared an Ap4A-biotin probe (Fig. 1) that bound with streptavidin-coated magnetic beads and found a suitable probe for the initial magnetic biopanning of cell lysates to identify prospective Ap4A binding proteins. The resulting magnetic beads of Ap4A-biotin were mixed with protein extracted from lyses of 100 μg/ml kanamycin-treated *E. coli* K12-MG1655, and after extensive washing, bound proteins were eluted with SDS sample buffer; the eluate fractions were subjected to SDS gel electrophoresis and stained with silver. As shown, several bands were detected in fractions from Ap4A-biotin magnetic beads of kanamycin-treated and untreated bacteria (Supplementary Fig. 1). These proteins were subjected to proteolytic digestion and MALDI-TOF–MS spectrometry. As a result, we identified several proteins that were unique in either kanamycin-treated or untreated strains (Fig. 2A). A total of 1240 proteins were identified by MALDI-TOF–MS proteomics, of which 204 different proteins were identified. Specifically, 166 proteins were released from Ap4A after kanamycin treatment, and 38 proteins bound to Ap4A after kanamycin treatment. Moreover, 1036 proteins were bound to both kanamycin-treated and untreated bacteria (Fig. 2B).

### Potential Ap4A binding proteins

**Fig. 3:**
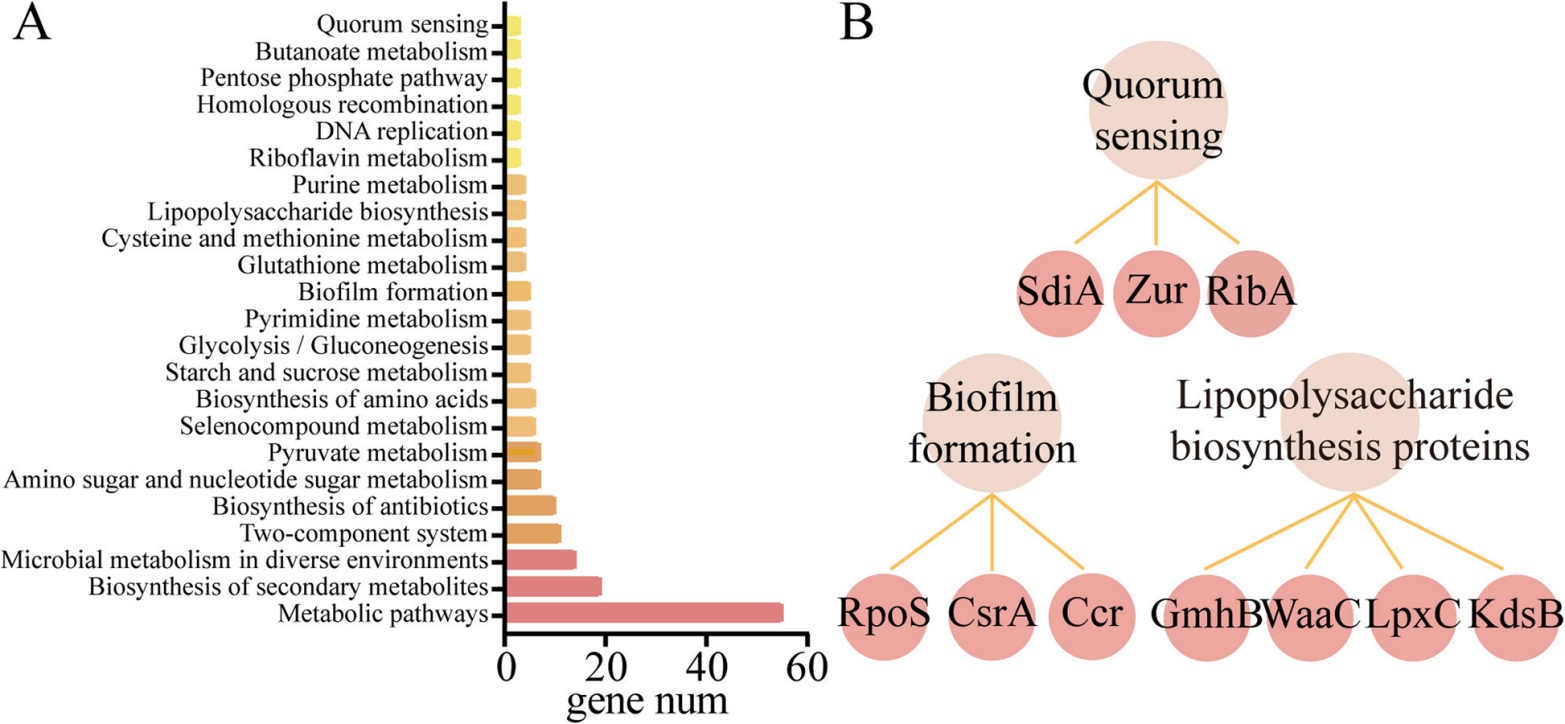
Enriched KEGG pathways of potential Ap4A-binding proteins following kanamycin treatment in *E. coli* K12-MG1655. **A** KEGG analysis of proteome data of Ap4A binding proteins. **B** The important enriched Ap4A-related pathways included biofilm formation, quorum sensing and lipopolysaccharide biosynthesis

To identify the key pathways altered by Ap4A in kanamycin-treated bacteria, we performed Gene Ontology (GO) analysis. GO analysis suggested that cytosol and cytoplasm pathways were enriched (Supplementary Fig. 2). Kyoto Encyclopedia of Genes and Genomes (KEGG) analysis showed that the ribosome biogenesis, peptidases and inhibitors, biofilm formation and quorum sensing pathways were regulated significantly (Fig. 3A). We used KEGG Brite analysis, and these pathways were correlated with biofilm formation proteins, including RpoS, CsrA and Ccr; quorum sensing proteins, including SdiA, Zur and RibA; and lipopolysaccharide biosynthesis proteins, including GmhB, WaaC, LpxC and KdsB (Fig. 3B). To explore the dynamic changes in the proteome of Ap4A binding proteins, we performed cluster and protein‒protein interaction (PPI) network analyses. Proteins in the cluster (*n* = 10) included RpoS, SdiA, CsrA, Uvr Y(CsrB), RelA(CsrC), LuxS, LpxD, LpxP, LpxC, and LpxA (Supplementary Fig. 3). These proteins were enriched in KEGG pathways, including biofilm formation, quorum sensing and lipopolysaccharide biosynthesis.

### Ap4A regulated biofilm formation, quorum sensing and lipopolysaccharide biosynthesis

**Fig. 4:**
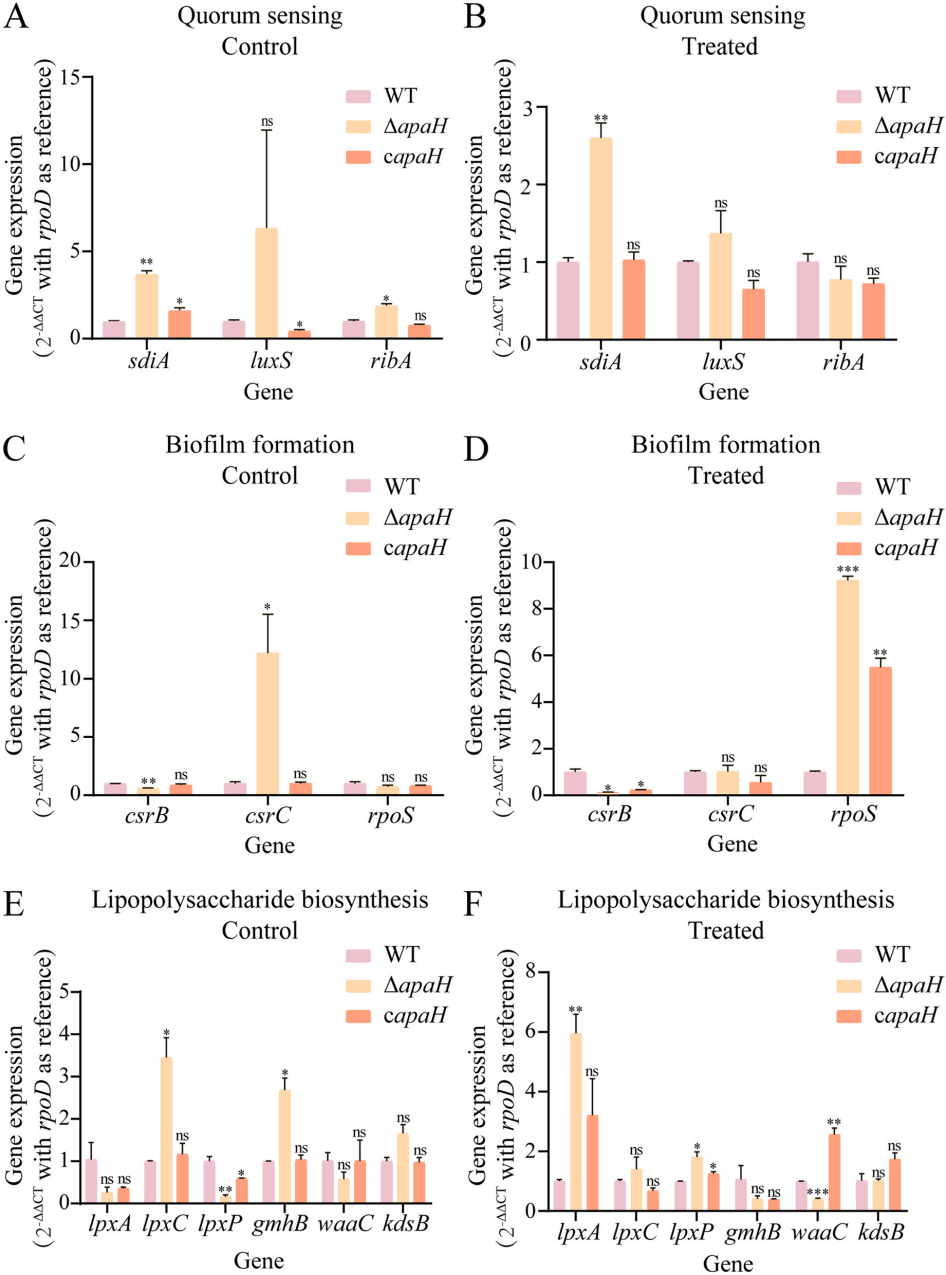
The effect of Ap4A on the expression of genes involved in quorum sensing, biofilm formation, and lipopolysaccharide biosynthesis in *E. coli* K12-MG1655. **A** The expression of genes in the quorum sensing pathway was affected by Ap4A; *rpoD* was used as a reference gene. **B** The expression of genes in the quorum sensing pathway was affected by Ap4A following kanamycin treatment; *rpoD* was used as a reference gene. **C** The expression of genes in the biofilm formation pathway was affected by Ap4A; *rpoD* was used as a reference gene. **D** The expression of genes in the biofilm formation pathway was affected by Ap4A following kanamycin treatment; *rpoD* was used as a reference gene. **E** The expression of genes in the lipopolysaccharide biosynthesis pathway was affected by Ap4A; *rpoD* was used as a reference gene. **F** The expression of genes in the lipopolysaccharide biosynthesis pathway was affected by Ap4A following kanamycin treatment; *rpoD* was used as a reference gene

To determine whether Ap4A regulated bacterial biofilm formation, quorum sensing and lipopolysaccharide biosynthesis pathways in kanamycin-treated bacteria, we measured the expression levels of important genes in these pathways in the wild-type strain and the *apaH* mutant strain, which had increased Ap4A. The results showed that when the wild-type and *apaH* mutant strains were not treated with kanamycin, the expression levels of quorum sensing genes, including *sdiA* and *ribA,* in the mutant strain significantly increased; expression of the biofilm formation gene *csrC* in the mutant strain was upregulated, and the expression of *csrB* was downregulated; and the expression of the lipopolysaccharide biosynthesis protein genes, including *lpxC* and *gmhB,* in the mutant strain were upregulated, and the expression of *lpxP* was downregulated (Fig. 4A, C and E). After kanamycin treatment, the expression of the quorum sensing gene *sdiA* in the *apaH* mutant was significantly upregulated; expression of the biofilm formation gene *rpoS* was upregulated, and the expression of *csrB* was downregulated; and the expression of lipopolysaccharide biosynthesis protein genes, including *lpxA* and *lpxP,* in the mutant strain were upregulated, and *waaC* expression was downregulated (Fig. 4B, D and F).

### Ap4A affected biofilm formation

**Fig. 5:**
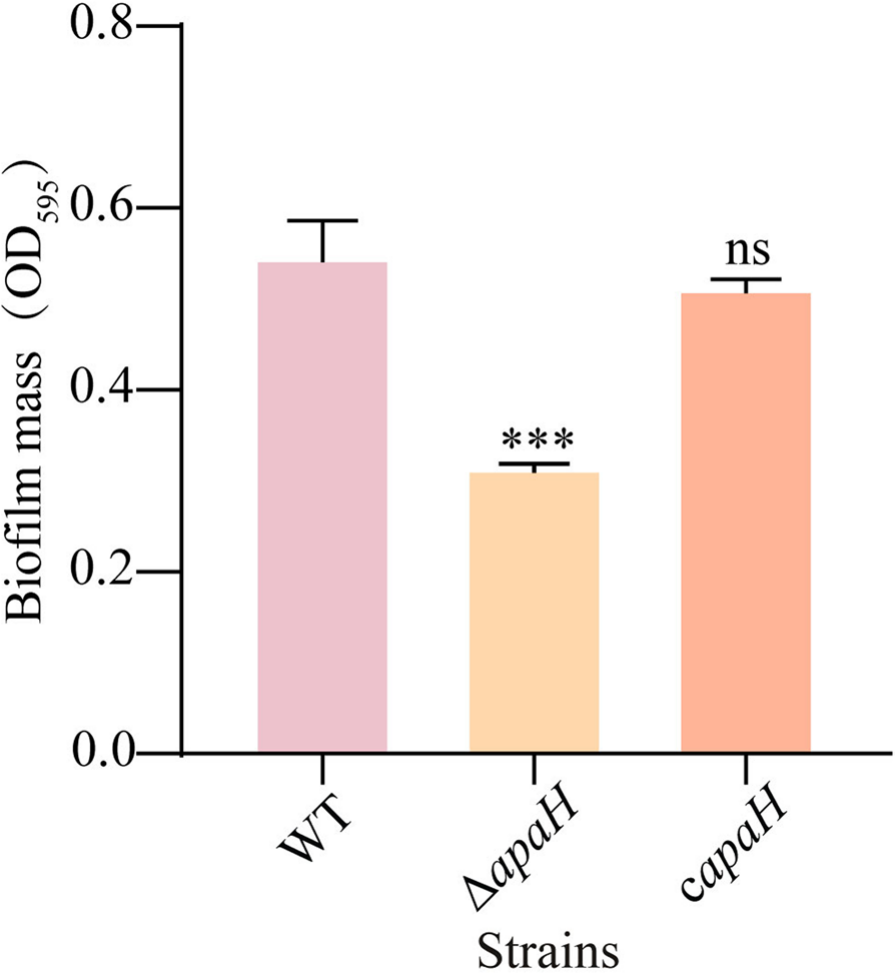
The role of Ap4A in *E. coli* K12-MG1655 biofilm formation. The biofilm of the wild-type MG1655 strains, the mutant strains Δ*apaH* and the complemented strains c*apaH* was tested on Luria–Bertani (LB) with kanamycin at the MIC (1.0728 × 10^–5^ M)

According to our previous results, we hypothesized that Ap4A increases bacterial sensitivity to kanamycin by regulating bacterial biofilm formation. Thus, we used the *apaH* mutant strain to increase the cellular Ap4A concentration and compared the biofilm formation of this strain to that of the wild type. The results showed that there was no difference in biofilm formation between the wild-type and *apaH* mutant strains (Supplementary Fig. 5), but we observed that the biofilm formation of the *apaH* mutant strain was significantly reduced after bacteria were treated with the minimum inhibitory concentration of kanamycin (1.0728 × 10^–5^ M) (Fig. 5 and Supplementary Fig. 4). The completed allele of the *apaH* gene in the mutant strain rescued biofilm formation under the same concentration of kanamycin treatment as the wild-type strain received (Fig. 5 and Supplementary Fig. 5).

### Ap4A affected swimming and swarming motility

**Fig. 6:**
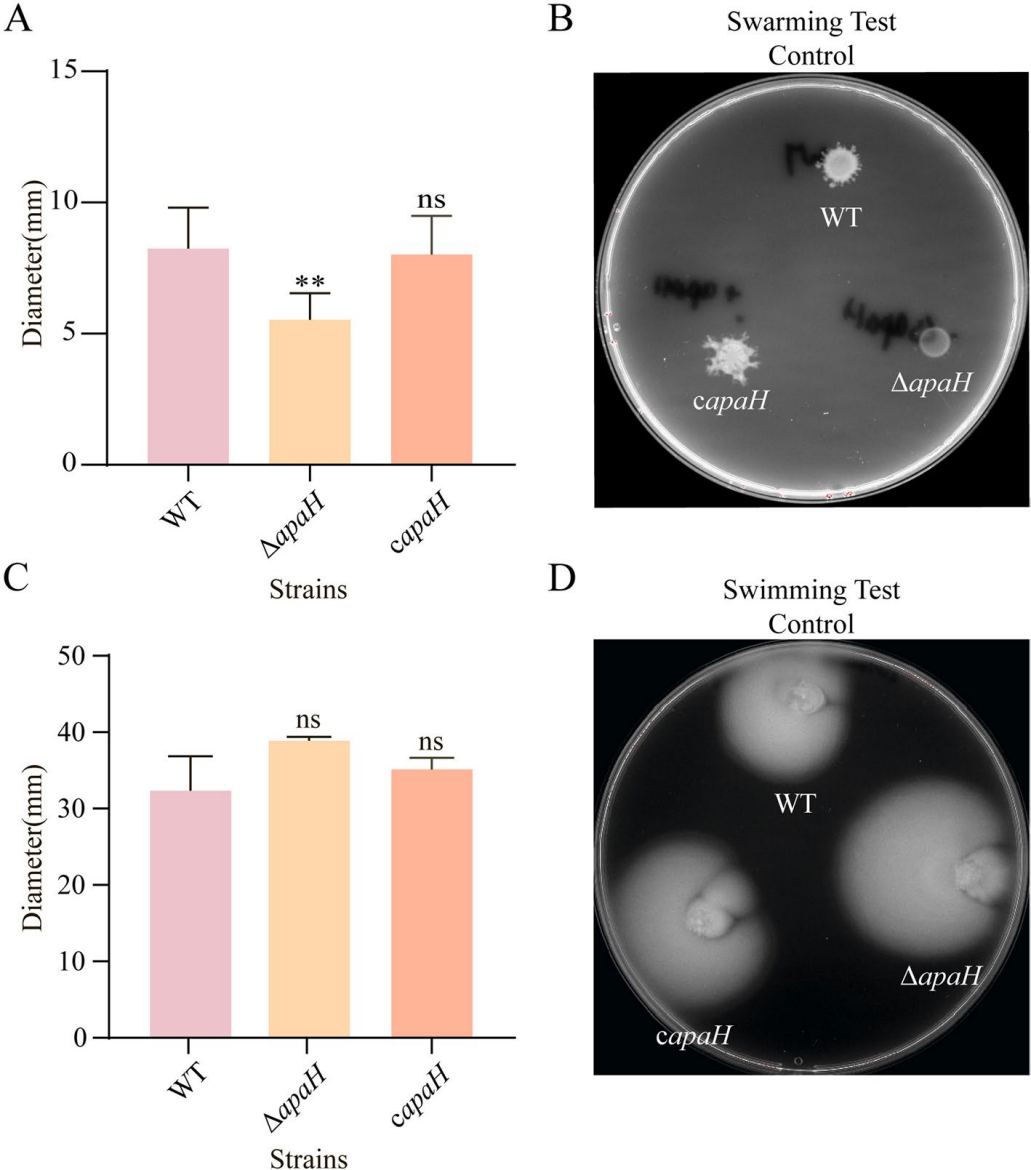
The role of Ap4A in *E. coli* K12-MG1655 swarming and swimming motilities. **A** The swarming motility diameter of the wild-type MG1655 strains, the mutant strains Δ*apaH* and the complemented strains c*apaH*. **B** The swarming motility of the wild-type MG1655 strains and the mutant strains Δ*apaH* was tested on Luria–Bertani (LB) plates containing 0.5% agar. **C** The swimming motility diameter of the wild-type MG1655 strains, the mutant strains Δ*apaH* and the complemented strains c*apaH*. **D** The swimming motility of the wild-type MG1655 strains and the mutant strains Δ*apaH* was tested on Luria–Bertani (LB) plates containing 0.3% agar

Bacterial motility is known to be linked to virulence and bacterial resistance to antibiotics. The swarming motility assay on soft agar plates showed that the *apaH*-defective mutant Δ*apaH* exhibited significantly decreased swarming motility compared to that of the wild-type strain (Fig. 6A-B), and this was inhibited more obviously when the *apaH* mutant strain was treated with kanamycin (Supplementary Fig. 6A-B). Partial recovery of swarming ability was observed in the *apaH* complement strain (Fig. 6A-B). However, the swimming motility of the wild-type and *apaH* mutant strains was not significantly different (Fig. 6C-D). When bacteria were treated with kanamycin, the swimming motility of both strains was inhibited (Supplementary Fig. 7A-B).

## Discussion

**Fig. 7:**
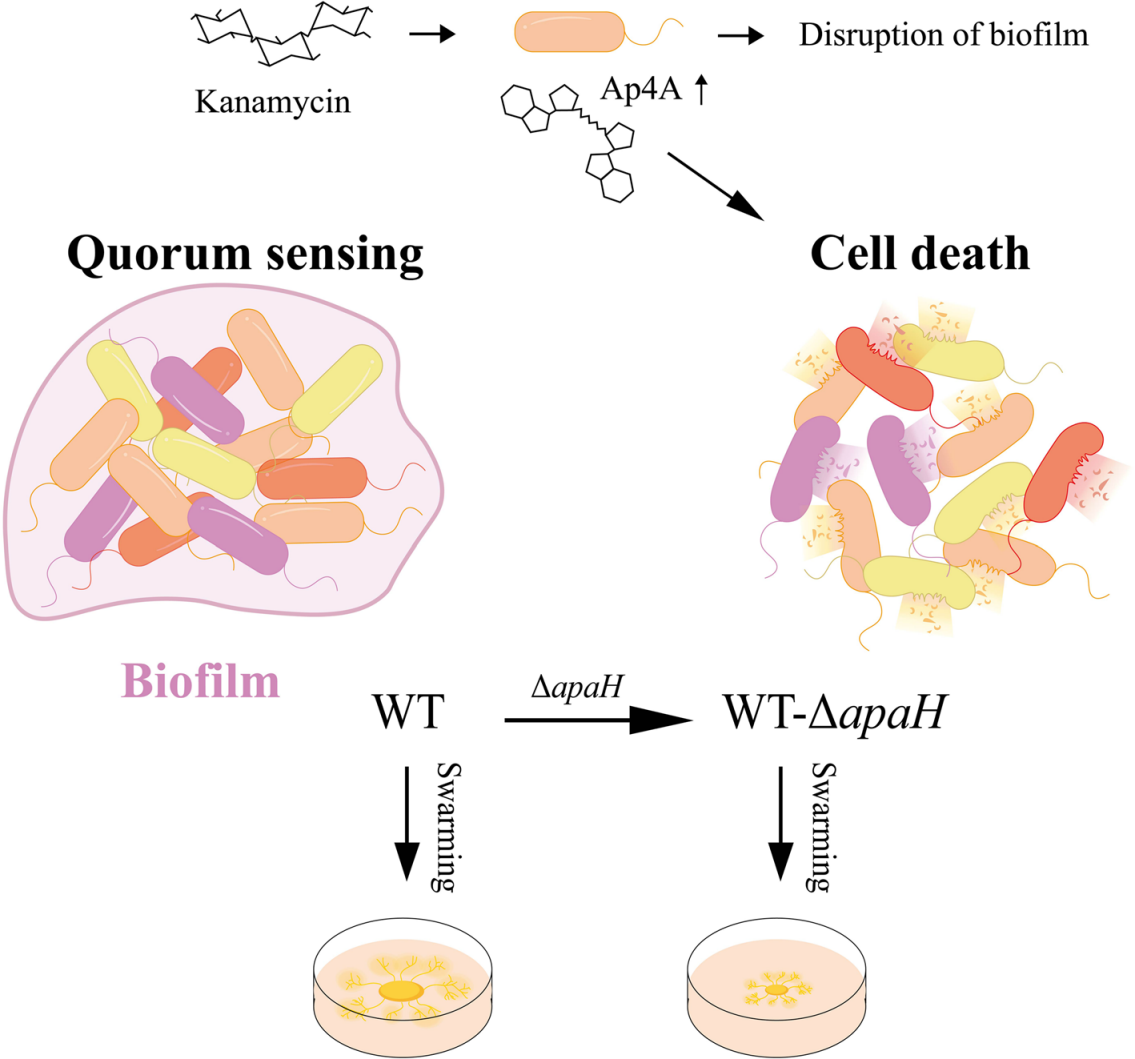
A model of Ap4A regulation of *E. coli* K12-MG1655 biofilm formation and swarming motility. The treatment of bacteria with kanamycin leads to Ap4A binding to quorum sensing related proteins to regulate bacterial biofilm formation and swarming motility, thereby promoting bacterial cell death

The treatment of bacterial infections associated with biofilms is extremely difficult [28]. In this paper, we found that Ap4A can regulate bacterial biofilm formation and quorum sensing to enhance the bactericidal effect of kanamycin. When bacteria were treated with kanamycin, Ap4A bound to 10 proteins (RpoS, CsrA, Ccr, SdiA, Zur, RibA, GmhB, WaaC, LpxC, KdsB) of three related signaling pathways, including the biofilm formation, quorum sensing, and lipopolysaccharide biosynthesis pathways (Fig. 3B). RpoS, RibA, and SdiA directly regulate the expression of LuxR family proteins that influence quorum sensing in bacteria [29–31]. RpoS is a key sigma factor that bacteria use to form biofilms and is responsible for guiding the transcription of many genes that are expressed during the stable period and under various stress conditions [32–39]. The activation of these genes makes bacteria more resistant to environmental stress, and the loss of the stress adaptation base RpoS significantly affects bacterial stress resistance [40]. LpxA, LpxD, LpxC and LpxP are related to lipopolysaccharide biosynthesis, biofilm formation [41–43], and LpxA/LpxD/LpxC complex lipid A biosynthesis; LpxA/LpxD/LpxC mutation can lead to changes in the composition of lipid A biosynthesis in cell membranes, which makes *Acinetobacter baumannii* resistant to polymyxin [44]. CsrA, RpoS, WaaC and GmhB can affect biofilm formation, virulence and bacterial motility to regulate the quorum sensing of bacteria [45–47]. Monds found that increased Ap4A in *Pseudomonas fluorescens* inhibited biofilm formation [15], and the gene expression of signaling pathways in the wild-type strain and the *apaH* mutant strain that had increased Ap4A was consistent with previous conclusions. In addition, compared with wild-type strains, the biofilm-forming ability of the *apaH* mutant strain was reduced under kanamycin treatment (Fig. 5). Thus, we suggest that when Ap4A accumulates in bacteria, it can alter biofilm formation and quorum sensing pathways to increase bacterial sensitivity to kanamycin (Fig. 7).

Antibiotic resistance is a global problem. Many common pathogenic bacteria have serious resistance to classical antibiotics, such as aminoglycosides and β-lactams, and the rate of resistance is increasing yearly [48]. The main mechanism of aminoglycoside antibiotics, such as kanamycin, is that they bind to the bacterial ribosome and lead to protein misfolding [49]. These misfolded proteins not only cause the loss of bacterial protein homeostasis but also insert into the cell membrane and disrupt the bacterial membrane structure [49]. Bacterial resistance to antibiotics is inevitable, and bacteria have quorum sensing and biofilms not only as a protective barrier against antibiotics [48], making it difficult for antibiotics to reach effective concentrations in internal bacteria [50], but also to assist in the swimming and swarming motility of bacterial flagella to increase bacterial virulence and pathogenicity [51]. Breaking the drug penetration barrier is an effective way to address bacterial resistance [52]. We found that Ap4A could reduce bacterial biofilm formation and affect quorum sensing when bacteria were treated with kanamycin. These results will help us identify compounds that can be used in combination with kanamycin and provide new ideas and methods to address bacterial resistance.

## Methods

### Bacterial strains and plasmids

The strains used in this study are listed in Table 1. LB broth was used for all experiments. The antibiotics kanamycin (Fisher Scientific, BP906-5) and ampicillin (Fisher Scientific, BP1760) were used for the experiments described in this study.

**Table 1:** Bacterial strains used in this study

Name	Description	Reference
*Escherichia coli* MG1655	Wild-type *Escherichia coli*, containing pBAD24 plasmid, amp^r^	[18]
*Escherichia coli* MG1655-Δ*apaH*	*Escherichia coli*; in-frame deletion of *apaH* (deleting amino acids from 1 to 280), containing pBAD24 plasmid, amp^r^	[18]
*Escherichia coli* MG1655-CΔ*apaH*	MG1655-Δ*apaH* containing pBAD24-MG1655*apaH* plasmid, amp^r^	[18]

### Preparation of biotin-labeled Ap4A

DMF was added to NH2-Reactive-Biotin, the concentration was adjusted to 10 mM, and the mixture was incubated for 10 min at room temperature. NH2-Reactive-Biotin (10 mM) was mixed with Ap4A in a tube and the sample was incubated for 30 min at 37 ℃. The mixture was added to the Millipore Ultacel tubes and centrifuged at 12,000 × g for 10 min at 4 ℃. The remaining buffer was biotin-labeled Ap4A. The Ap4A-biotin concentration was measured with a colorimetric biotin assay kit (sigma, MAK171). Briefly, 20 μl of each biotin-containing sample, the negative control (ultrapure water), and the positive control (MAK171C) were added to the appropriate wells of a 96-well white/clear bottom microplate. 180 μl of HABA/avidin assay mixture was added into each well of the biotin-containing samples, the negative control, and the positive control to make the total assay volume 200 μl/well. The reaction mixture was incubated at room temperature for 5 min on a plate shaker at 100–200 rpm in the dark. The absorbance was monitored with a plate reader at 500 nm (A_500_).

$$\mathrm{Calculations as Ap}4\mathrm{A}-\mathrm{biotin concentration }(\mathrm{M})=\frac{\left({\mathrm{A}}_{500}\mathrm{ negative control}-{\mathrm{A}}_{500}\mathrm{ biotin sample or positive control}\right)\times \mathrm{dilution factor}}{\left(34, 500\times 0.5\right)}$$

### Affinity purification of Ap4A-binding proteins

30 ml of fresh *E. coli* K12-MG1655 bacterial cultures were grown at 37 ℃ until the exponential phase and treated with 100 μg/ml kanamycin for 1 h. Bacteria without kanamycin treatment were used as controls. Total proteins were extracted from the bacterial lysates according to the manufacturer’s instructions (Sangon Biotech, C600596-0001). The protein concentration of the extracts was determined with a Micro BCA Protein Assay (Sangon Biotech, C503061-1250). Biotin binding beads were equilibrated with PBS (136.89 mM NaCl; 2.67 mM KCl; 8.1 mM Na_2_HPO_4_; 1.76 mM KH_2_PO_4_). 20 μl of 47.4 μM Ap4A-Biotin was incubated with 0.4 mg of beads for 30 min. The beads were washed twice with B&W buffer (5 mM Tris–HCl pH = 8.0, 0.5 mM EDTA, 1 M NaCl) and resuspended in 100 μl Tris–HCl buffer (50 mM Tris–HCl, 100 mM NaCl, 10 mM MgCl_2_, 10 mM KCl, pH = 8.0). Extract proteins were incubated with prepared Ap4A-biotin binding beads for 30 min. The beads were washed three times with PBS and resuspended in 50 μl Tris–HCl buffer. Eluted samples were separated by SDS‒PAGE and detected by silver.

### Identification of Ap4A-binding proteins

The Ap4A-bound proteins were sent to Beijing Qinglian Boao Biotechnology for LC‒MS/MS. Briefly, the proteins were subjected to trypsin digestion. Peptides were analyzed with a matrix-assisted laser desorption ionization time-of-flight mass spectrometer (MALDI-TOF–MS, Bruker Daltonics, MI, USA) coupled with high-performance liquid chromatography (LC–MS/MS). The samples were analyzed with the following settings: Spray voltage: 2200 V; Capillary temperature: 350 °C; Ion Source: NSI; Full MS: Resolution: 120000FWHM; Full Scan AGC target: 2e^5^; Full Scan Max.IT: 100 ms; Scan range: 300–1400 m/z; dd-MS2: AGC target: 5e^3^; Maximum IT: 35 ms; and NCE: 32%. Data were analyzed by Proteome Discoverer 2.4 to identify proteins corresponding to each peak.

### Bioinformatics analysis

We collected data from the following sources: UniProt_*E. coli* was used to collect the proteomes; the UniProt *E. coli* data were 2020.08.13 download (4391 sequence) with the following rules: static modification: C carboxyamidomethylation, dynamic modification: oxidation (M), and false discovery rate ≥ 0.01. Proteins unique sequence screening was performed as follows: score sequest HT: sequest HT > 0, and unique peptides ≥ 1. Pathway and biological function enrichment of clusters was performed using DAVID 6.8 (https://david.ncifcrf.gov/) and the Kyoto Encyclopedia of Genes and Genomes (KEGG, www.kegg.jp/). Cytoscape 3.8.2 and STRING 11.0 (https://www.string-db.org/) plug-ins were used for visual analysis of the protein‒protein interaction network.

### Bacterial RNA extraction and qRT‒PCR

Overnight cultured bacteria were diluted 1:100 in LB medium with 0.02% arabinose and grown until OD_600_ = 0.5. Then, the bacteria were treated with 100 μg/ml kanamycin for 30 min. The treated bacteria were harvested, and the total RNA was extracted from bacteria using a bacterial RNA extraction kit (onrew, RNB481-02). Total RNA (1 μg) was reverse-transcribed to cDNA using the Prime Script™ RT Reagent Kit with gDNA Eraser Kit (TaKaRa, RR047Q). qRT‒PCR was performed on 10 ng cDNA using the ChamQ SYBR Color qPCR Master Mix (Low ROX Premixed) kit (Vazyme, Q431) in a StepOnePlus real-time PCR system (Applied Biosystems) with a two-step method as follows: initial denaturation at 95 ℃ for 30 s; 40 cycles of 95 ℃ for 10 s and 60 ℃ for 30 s. The transcription level of each gene was normalized to that of the reference gene, *rpoD* [53]. The primers utilized are listed in Table 2.

**Table 2:** Primers used in this study

Primers	Sequences(5' to 3')
*rpoD*-F	TCTGCGTATGCGTTTCGGTATC
*rpoD*-R	ACGGCTCGGGTGACGCAGTT
*rpoS*-F	CATAATCGCCCGTTCAATCG
*rpoS*-R	CGTTATGGCAATCGTGGTCTG
*ribA*-F	TTACGACCTTCCTGACGGTGAT
*ribA*-R	CCTGATGGTGGGATTTGAAGAAC
*csrB*-F	CGGTTCGTTTCGCAGCATTC
*csrB*-R	GGCCAGGGACACTTCAGG
*csrC*-F	GGACGCTAACAGGAACAATGAC
*csrC*-R	CCTTAACGGGTCTTACAATCCTTGC
*sdiA*-F	GATAATAACTAACCCACGCCTCAGG
*sdiA*-R	GAGGATGGAGACCGCAGAAGA
*luxS*-F	GGATCTGATTCTGATCCTGCACTT
*luxS*-R	ACCCTGGAGCACCTGTTTG
*lpxA*-F	GAGATTTATCAGTTCGCCTCCATCG
*lpxA*-R	TGCCCACCTTCGTCAATCCAC
*lpxC*-F	AAGATGGCGATAAGTGGGCTGA
*lpxC*-R	CACGCATGAAACCGAACGT
*gmhB*-F	CATCATCCGCAGGGTAGTGTTG
*gmhB*-R	CAGGCGTAATAGGTTTACCCG
*lpxP-*F	GGTTTGATGTTGAAGGGTTGGA
*lpxP-R*	CCATCAGCTGATTATTATGTGGACG
*waaC-*F	AGAACGGGCGAAACGACT
*waaC-R*	CTGATTCTTCCCATACCCACCAA
*kdsB-F*	GTGGGTATGGCGACTCTGG
*kdsB-R*	ACGTAACGACGGATAAAGCCTG

### Biofilm formation assay

*E. coli* K12-MG1655, *E. coli* K12-MG1655-Δ*apaH* and *E. coli* K12-MG1655-Δ*apaH*-*apaH* were incubated overnight at 37 ℃ in LB and diluted to OD_600_ = 0.02. 150 μl of diluted culture was added into a 96-well microtiter plate, and the tests were performed in three replicates. After incubation for 24 h, the plates were washed with distilled water and then dried. After the drying step, 180 μl of 0.1% crystal violet solution was added to each well. The plates were incubated at room temperature for 15 min and washed three times. After drying, 30% glacial acetic acid was added to the wells, and the absorbance was measured at 595 nm in a microplate reader (Bio Tek, Synergy H1).

### Bacterial motility assays

To perform the swarming motility assay, overnight fresh bacterial culture samples were diluted to OD_600_ = 1.0 in a 1.5 ml tube containing 1 ml LB. 5 μl samples at OD 1 were spotted onto the center of LB soft agar plates (0.5% [w/v] agar, 25 g/liter LB broth, 5 g/L glucose). The plates were then incubated for 48 h at 37 ℃ and photographed, and the radius of the swarming was measured based on the average dendrite lengths.

To perform the swimming motility assay, overnight fresh bacterial culture samples were diluted to OD_600_ = 1.0 in a 1.5 ml tube containing 1 ml LB. Samples were tipped into the center of LB soft agar plates (0.3% [w/v] agar, 25 g/liter LB broth). The plates were then incubated for 24 h at 37 ℃ and photographed, and the radius of the swimming was measured based on the average dendrite lengths.

### Statistical analyses

Statistical analyses were performed by the GraphPad Prism 8 analyses program with a *t* test to identify significant differences between samples and the control group. All experiments were performed in triplicate. Data are presented as the mean; biofilm error margins are presented as standard error (SE). *P* value below 0.05 was considered statistically significant.

### Supplementary Information

**Additional file 1:** **Supplementary Figure 1.** Screening Ap4A binding candidate proteins. Silver stain of the Ap4A binding candidate proteins after Ap4A-biotin immunoprecipitation (IP) with the protein lysate of 100 μg/ml kanamycin treatment or not treatment with *E. coli* K12-MG1655. **Supplementary Figure2.** Gene ontology (GO) analysis of proteome data from Ap4A binding proteins under kanamycin treatment *E. coli* K12-MG1655. **Supplementary Figure 3.** The protein-protein interaction (PPI) network analyses of Ap4A binding proteins in *E. coli* K12 MG1655. **Supplementary Figure 4.** The kanamycin MIC of the wild-type, mutant strain Δ*apaH*, and complemented strain were tested on Luria-Bertani (LB) medium. **Supplementary Figure5.** The biofilm formation of the wild-type, *apaH* mutant, and complemented strains without kanamycin treatment. **Supplementary Figure 6.** The swarming motility of wild-type, *apaH* mutant and complemented strains with kanamycin treatment. (A)The swarming motility diameter of wild-type, *apaH* mutant, and complemented strains*. *(B)The swarming motility of wild-type, *apaH* mutant, and complemented strains were tested on Luria-Bertani (LB) plates containing 10 μg/ml kanamycin, 0.5% agar. **Supplementary Figure 7.** The swimming motility of wild-type, *apaH* mutant, and complemented strains with kanamycin treatment. (A)The swimming motility diameter of wild-type, *apaH* mutant, and complemented strains*. *(B) The swimming motility of wild-type, *apaH* mutant, and complemented strains were tested on Luria-Bertani (LB) plates containing 10 μg/ml kanamycin, 0.3% agar.

## Data Availability

The authors state that data supporting the findings of the study are included in the article and that materials are available upon request.

## Abbreviations

Ap4A: Diadenosine tetraphosphate
LCMS: Liquid chromatography-mass spectrometry
QS: Quorum sensing
GO: Gene Ontology
KEGG: Kyoto Encyclopedia of Genes and Genomes
PPI: Protein–protein interaction
PBS: Phosphate-buffered saline
SE: Standard error
