# Supplementary material for: Diadenosine tetraphosphate modulated quorum sensing in bacteria treated with kanamycin
Source: BMC Microbiol. 2023 Nov 17;23:353. doi: 10.1186/s12866-023-03113-3 (PMC10657157; doi:10.1186/s12866-023-03113-3)
Supplement: Supplementary file 1 — Additional file 1: Supplementary Figure 1. Screening Ap4A binding candidate proteins. Silver stain of the Ap4A binding candidate proteins after Ap4A-biotin immunoprecipitation (IP) with the protein lysate of 100 μg/ml kanamycin treatment or not treatment with E. coli K12-MG1655. Supplementary Figure2. Gene ontology (GO) analysis of proteome data from Ap4A binding proteins under kanamycin treatment E. coli K12-MG1655. Supplementary Figure 3. The protein-protein interaction (PPI) network analyses of Ap4A binding proteins in E. coli K12 MG1655. Supplementary Figure 4. The kanamycin MIC of the wild-type, mutant strain ΔapaH, and complemented strain were tested on Luria-Bertani (LB) medium. Supplementary Figure5. The biofilm formation of the wild-type, apaH mutant, and complemented strains without kanamycin treatment. Supplementary Figure 6. The swarming motility of wild-type, apaH mutant and complemented strains with kanamycin treatment. (A)The swarming motility diameter of wild-type, apaH mutant, and complemented strains. (B)The swarming motility of wild-type, apaH mutant, and complemented strains were tested on Luria-Bertani (LB) plates containing 10 μg/ml kanamycin, 0.5% agar. Supplementary Figure 7. The swimming motility of wild-type, apaH mutant, and complemented strains with kanamycin treatment. (A)The swimming motility diameter of wild-type, apaH mutant, and complemented strains. (B) The swimming motility of wild-type, apaH mutant, and complemented strains were tested on Luria-Bertani (LB) plates containing 10 μg/ml kanamycin, 0.3% agar. [file 12866_2023_3113_MOESM1_ESM.docx]

Diadenosine tetraphosphate modulated quorum sensing in bacteria treated with kanamycin

**Xia Ji^1#*^, Ruojing Yu^1#^, Meilian Zhu^1^, Cuilin Zhang^1^, Libin Zhou^l^, Tianshu Cai^2^, Weiwei Li^2^**

^1^School of Life Science, Huizhou University, Huizhou, 516007, China

^2^Huizhou Health Sciences Polytechnic, Huizhou, 516025, China

# These authors contributed equally to this work

*** Correspondence:**

Xia Ji: [xiaji@hzu.edu.cn](mailto:xiaji@hzu.edu.cn)

**Keywords:** kanamycin, diadenosine tetraphosphate, biofilm, *apaH*, quorum sensing

**Running Title: Ap4A Regulated Bacterial** **Quorum Sensing**


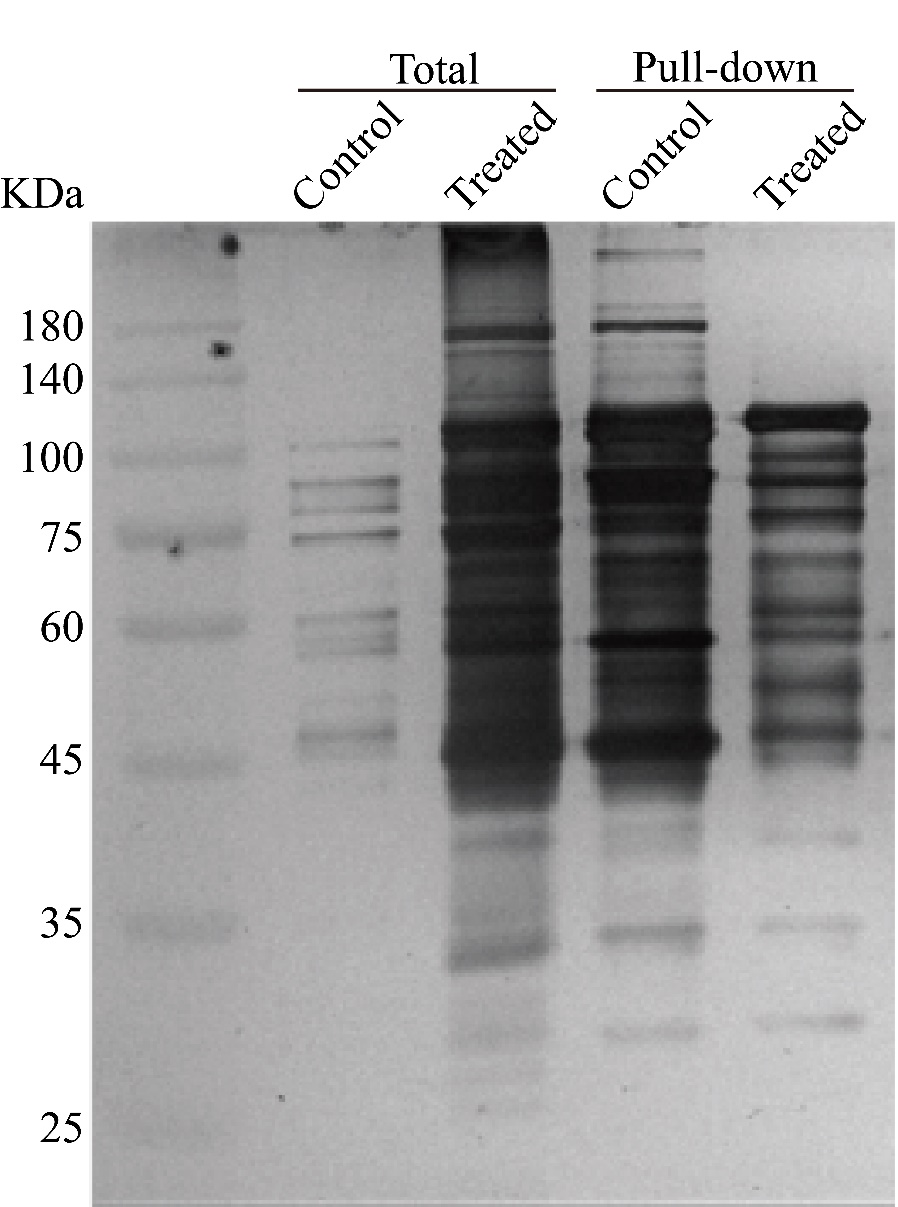


**Supplementary Figure 1. Screening Ap4A binding candidate proteins.** Silver stain of the Ap4A binding candidate proteins after Ap4A-biotin immunoprecipitation (IP) with the protein lysate of 100 μg/ml kanamycin treatment or not treatment with *E. coli* K12-MG1655.


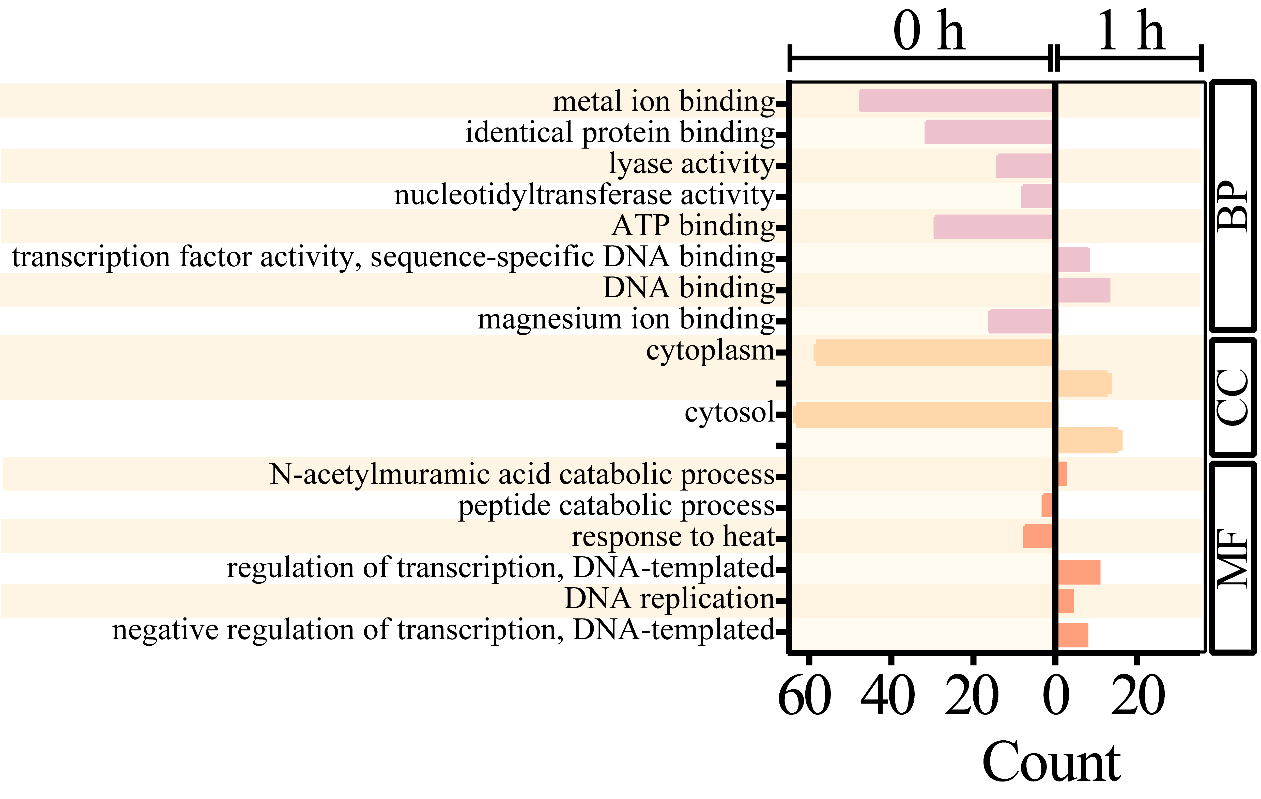


**Supplementary Figure 2.Gene ontology (GO) analysis of proteome data from Ap4A binding proteins under kanamycin treatment *E. coli* K12-MG1655.**


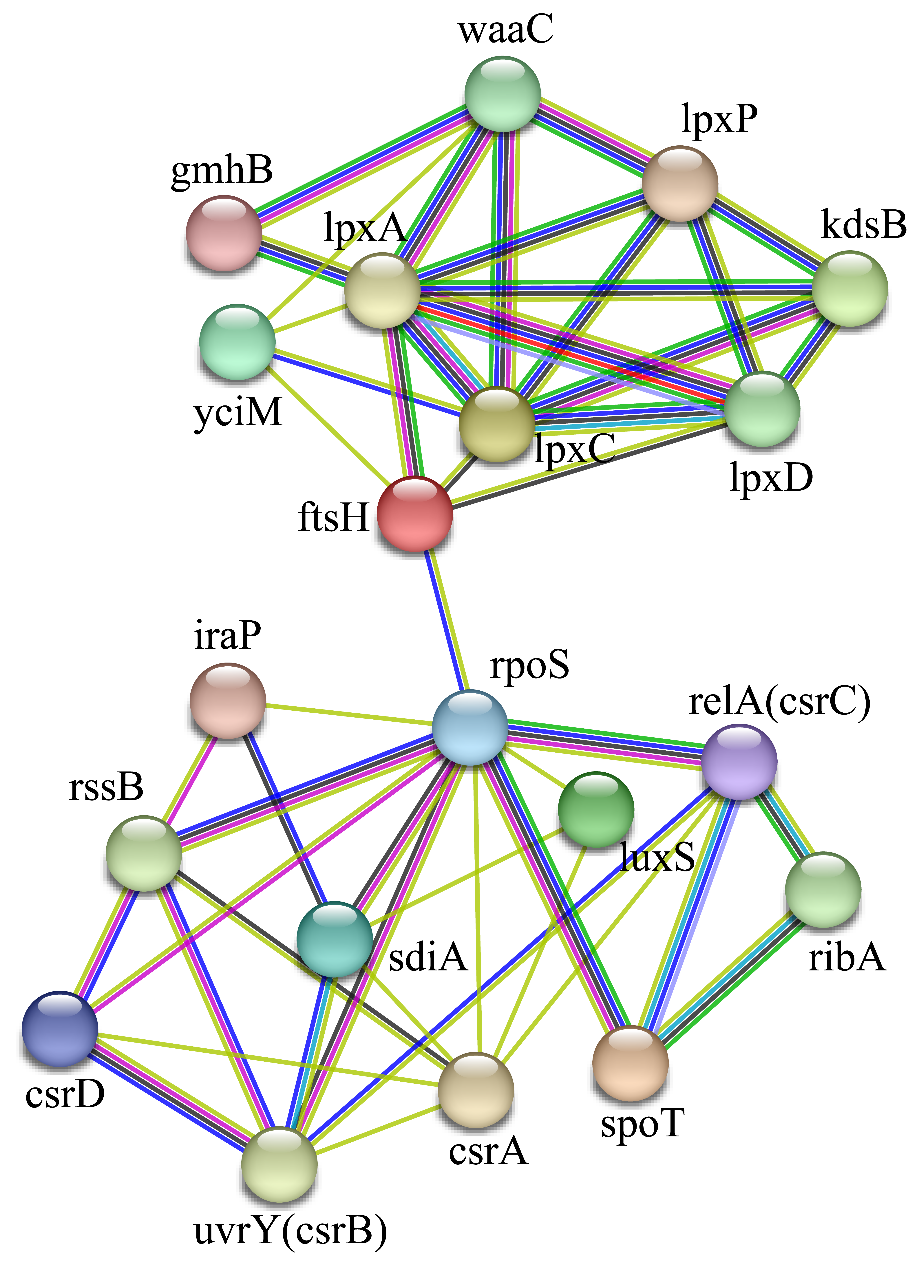


**Supplementary Figure 3.The protein-protein interaction (PPI) network analyses of Ap4A binding proteins in *E. coli* K12 MG1655.**


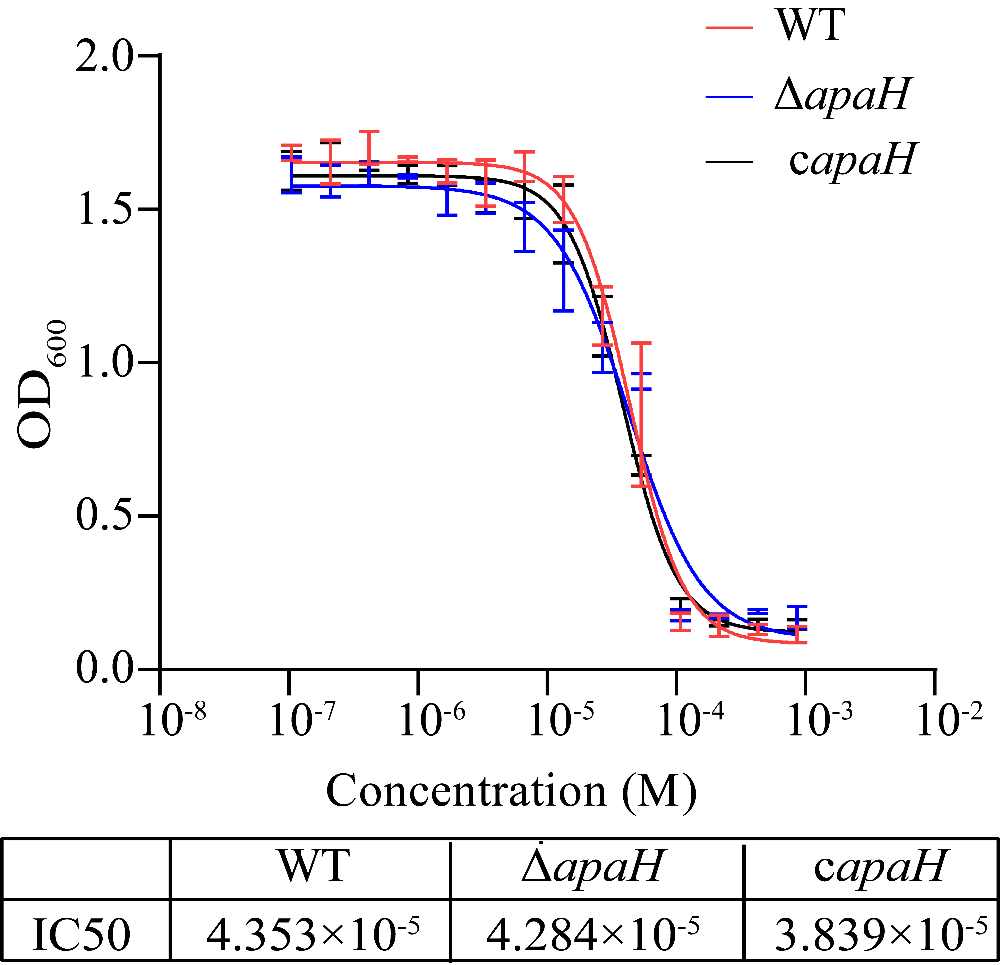


**Supplementary Figure 4. The kanamycin MIC of the wild-type, mutant strain Δ*apaH*, and complemented strain were tested on Luria-Bertani (LB) medium.**


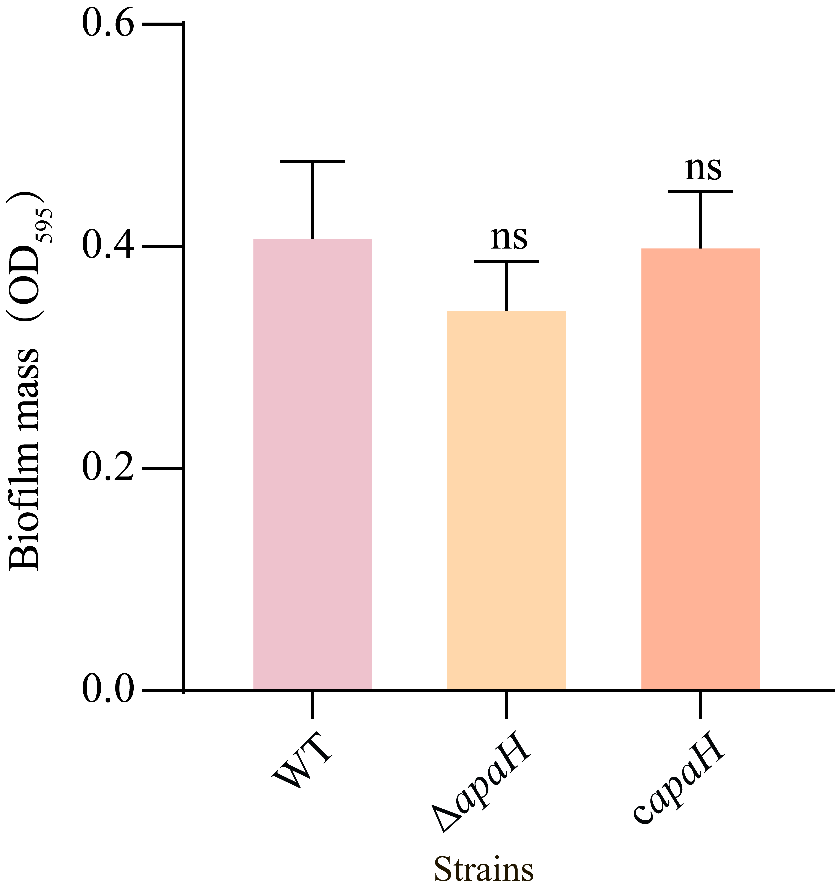


**Supplementary Figure 5. The biofilm formation of the wild-type, *apaH* mutant, and complemented strains without kanamycin treatment**.


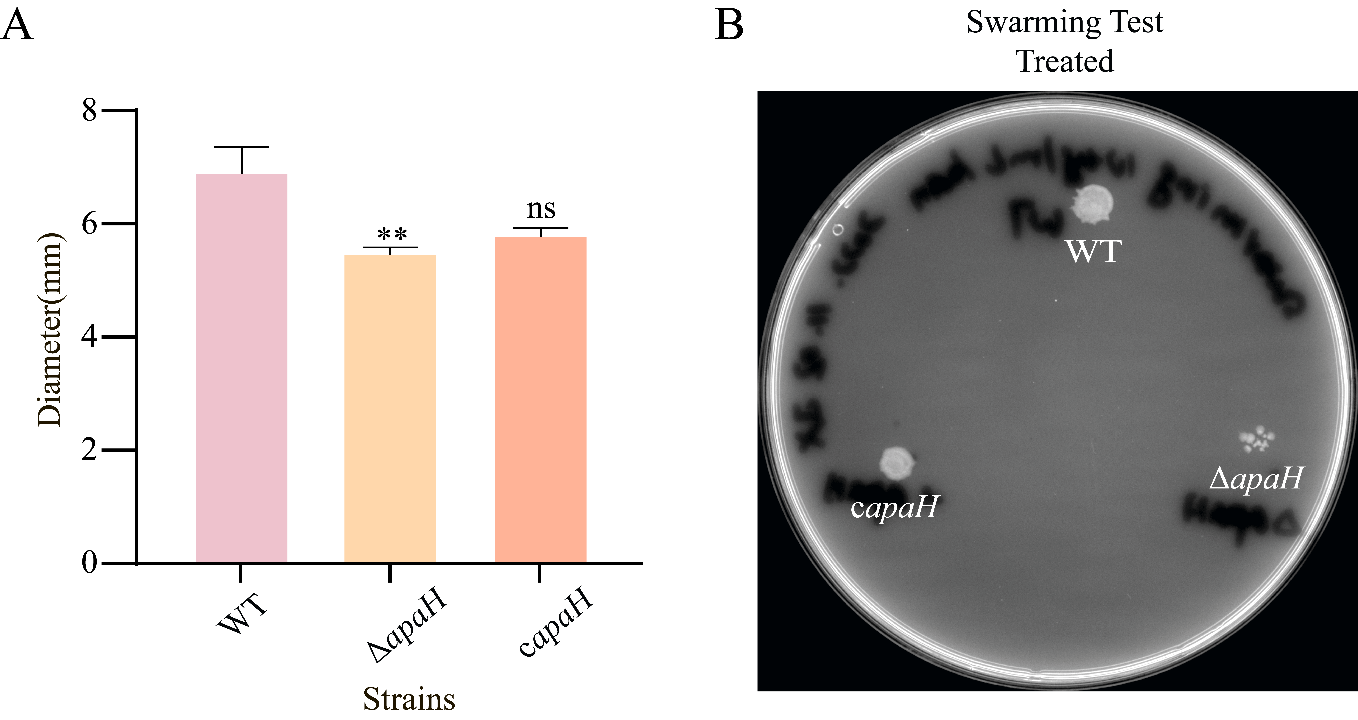


**Supplementary Figure 6. The swarming motility of wild-type, *apaH* mutant and complemented strains with kanamycin treatment.** (A)The swarming motility diameter of wild-type, *apaH* mutant, and complemented strains*.* (B)The swarming motility of wild-type, *apaH* mutant, and complemented strains were tested on Luria-Bertani (LB) plates containing 10 μg/ml kanamycin, 0.5% agar.


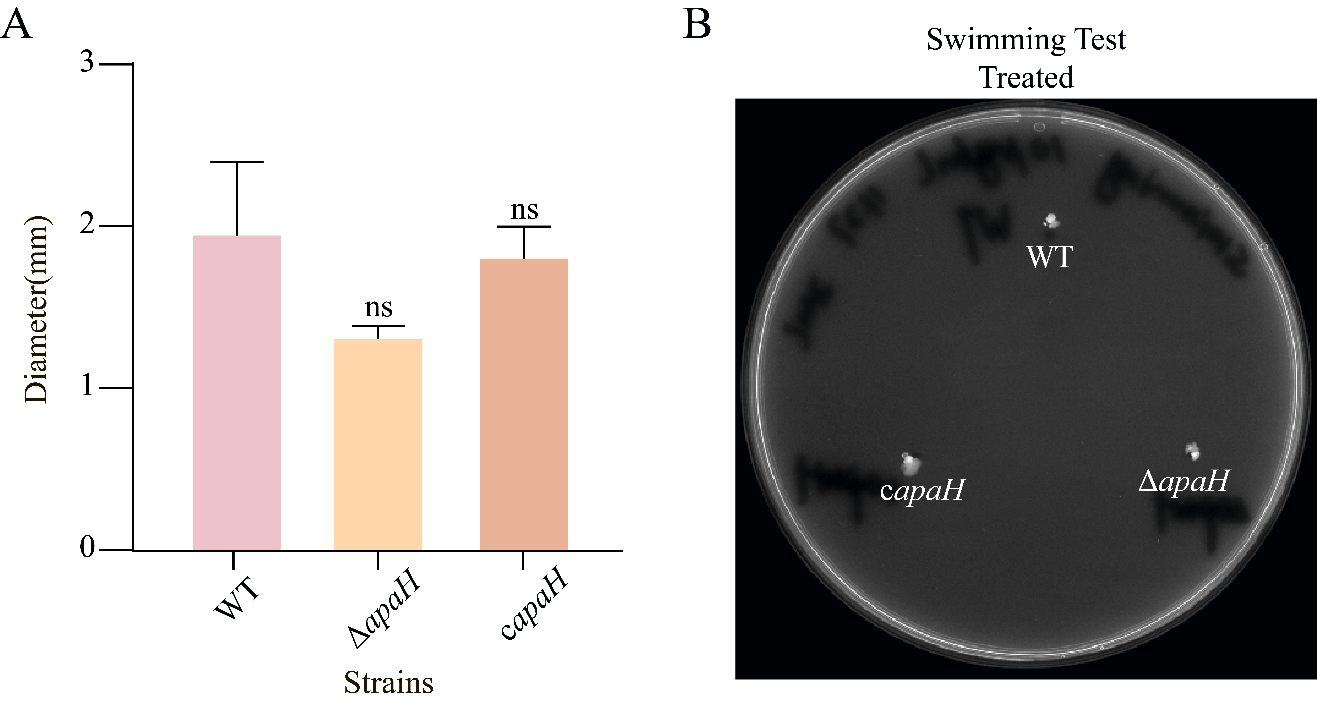


**Supplementary Figure 7. The swimming motility of wild-type, *apaH* mutant, and complemented strains with kanamycin treatment.** (A)The swimming motility diameter of wild-type, *apaH* mutant, and complemented strains*.* (B) The swimming motility of wild-type, *apaH* mutant, and complemented strains were tested on Luria-Bertani (LB) plates containing 10 μg/ml kanamycin, 0.3% agar.
